# A shock to the (health) system: experiences of adults with rare disorders during the first COVID-19 wave

**DOI:** 10.1186/s13023-024-03033-z

**Published:** 2024-01-30

**Authors:** Kathleen R. Bogart, Annelise Hartinger, Maggie Klaus, Elizabeth Jenkinson

**Affiliations:** 1 School of Psychological Science, Oregon State University, 2950 SW Jefferson Way, Corvallis, OR 97331 USA; 2 Centre for Appearance Research, University of the West of England, Bristol, UK

**Keywords:** Rare disease, Rare disorder, Coping, Social support, Healthcare access, COVID-19

## Abstract

**Background:**

Before COVID-19, people with rare diseases (RD) experienced numerous disparities in quality of life and healthcare access and quality, yet little is known about the experiences of this underserved group during the pandemic.

**Results:**

During the first wave of the COVID-19 pandemic in the United States, spring and summer of 2020, 759 participants representing 231 unique RDs responded to open-ended questions about the impact of the pandemic on life with a RD, healthcare access, and coping. Qualitative conventional content analysis was used to analyze responses. Identified themes represented positive and negative dimensions of change, including *a shock to the (health) system*, *coping with uncertainty*, and *the value of social support while isolated*.

**Conclusions:**

Limitations in healthcare access and quality were the most frequently described as impacts of COVID-19. Other major negative impacts included exacerbation of symptoms, psychological distress, and a lack of usual social support and reliable information. However, participants also noted silver linings, especially in healthcare. For some, expanded telehealth enhanced their ability to access medical and mental health providers and RD specialists. Finally, many participants hoped that, by highlighting social and health inequities faced by people with RDs and other minorities, the pandemic would prompt greater understanding and policies that could improve the quality of life of the RD community.

**Supplementary Information:**

The online version contains supplementary material available at 10.1186/s13023-024-03033-z.

## Introduction

A rare disease or disorder (RD) is defined in the United States (US) as a condition that affects fewer than 200,000 people [1], and in Europe as a condition that affects fewer than 1 in 2000 [2]. While an individual RD is uncommon, the prevalence of RDs as a whole is not. Currently, there are approximately seven thousand recognized RDs [2]. An estimated 3.5–10 percent of the population lives with a RD [1, 3]. Each RD presents its own unique challenges, but members of the RD community face many similar problems that arise from having a condition that is not well understood [4].

Most RDs are severe, chronic, and affect multiple systems [5]. In the US, the average individual with a RD waits 9 years for a correct diagnosis [6]. Those with RDs have lower health-related quality of life compared to the general population, and to those with common chronic disorders [6, 7]. Major causes of this decreased quality of life are difficulty getting a diagnosis, limited RD information, a lack of treatment options, limited psychosocial support, and social stigma [4]. Given these existing social and health disparities involved with living with a RD, the COVID-19 pandemic may have had a particularly detrimental effect on this community.

While some studies highlighted the negative impacts on continuity and quality of healthcare in a specific RD types [8–10], fewer have examined the experiences of the RD community as a whole during the pandemic. The National Organization for Rare Disorders conducted a quantitative survey of adults with RDs and caregivers primarily from the US [11]. They found that 79% of their participants had a medical appointment canceled because of the pandemic, and 62% were concerned about medical supply shortages. Thirty-seven percent had been impacted by loss of income. This study highlights major healthcare access issues, employment/financial concerns, and increased risk of COVID-19 in the RD population.

Similarly, a qualitative study of representatives from German patient support organizations found that the pandemic intensified challenges experienced by people with RD and their caregivers, including disruption of healthcare and support networks [12]. Patient organizations were a crucial provider of information and social support. There was an increase in telehealth provision of physical and mental healthcare, yet there was a remaining need for routine psychosocial support.

In a qualitative study, Halley and colleagues [13] surveyed people with a variety of rare and undiagnosed diseases and their caregivers living in America during the pandemic. They found six themes: barriers to accessing essential health care; impacts of COVID-19 visitation policies on their ability to advocate in health care settings; uncertainty and fear regarding COVID-19 infection risk; physical and mental health challenges; impacts of reduced educational and therapeutic services; and unexpected positive changes. Results indicated that the pandemic exacerbated longstanding challenges around diagnostic and treatment delay, and created new challenges. For example, due to the complexity of RDs, many people with RDs rely on caregivers to advocate for them during medical appointments, yet COVID-19 precautions often restricted the ability of the caregiver to be present and support their care.

Because these previous studies focused on healthcare access, they provide valuable insight into an alarming set of barriers and challenges experienced by those with RDs during the pandemic. However, previous research was not designed to capture psychosocial experiences. Our current study extends previous work with a qualitative approach to understanding the experiences of adults with RDs in the U.S. during the early months of the COVID-19 pandemic, including challenges related to psychosocial issues and access to healthcare and treatment.

## Method

### Participants

Participants were recruited from US-based RD organizations including Coordination of Rare Diseases at Sanford, social media, and previous RD study participant contact lists. Although the focus was on US experiences, English-speaking international participants were permitted to enroll. Researchers confirmed self-reported disorders were rare according to the NIH definition using their Genetic and Rare Disorder Information Center database [14]. A total of 759 participants responded to the questions of interest and were thus included in the study. See participant characteristics in Table 1. Participants were adults with at least one RD, however 212 (27.93%) of our participants had 2 or more RDs. There were 231 RDs represented among participants, the most common being spinocerebellar ataxia (10.3%), idiopathic hypersomnia (6.4%), Ehlers Danlos syndrome (5.7%), mast cell activation syndrome (5.5%), and narcolepsy (3.7%). The average age of participants was 52.03 years old (SD = 15.39) and 75.23% were female. Most participants, 649 (85.51%), lived in the US when participating in our study, while less than 1% each resided in other countries including Canada, United Kingdom, Spain, Germany, Ireland, the Netherlands, Switzerland, Belgium, France, India, Italy, Mexico, New Zealand, Norway, Portugal, and South Africa.

**Table 1:** Participant demographics

*Gender*	
Female	75.23%
Male	24.11%
Other	0.01%
*Age*	
18–29	8.43%
30–49	33.20%
50–64	34.39%
65 +	23.98%
*Income*	
Under $10,000	3.95%
$10,000–$20,000	5.93%
$20,001–$30,000	8.04%
$30,001–$45,000	10.67%
$45,001–$60,000	10.94%
$60,001–$75,000	9.62%
$75,001–$90,000	10.28%
$90,000 +	35.97%
Didn’t Answer	4.61%
*Race*	
White	90.38%
Hispanic or Lantino/a	0.02%
Asian	0.02%
American Indian or Alaskan Native	0.01%
Black or African American	0.00%
Native Hawaiian or Pacific Islander	0.00%
Other	0.02%

### Procedure

Participants followed a link to the survey administration platform Qualtrics. Data collection occurred between May 6, 2020 and continued for 10 weeks, ending on July 15, 2020. The current paper reports on responses to 9 open-ended questions concerning experiences during the COVID-19 pandemic, healthcare access, coping, silver linings, and cultural or societal changes that participants hoped would result. Full questions are available as Additional file 1.

### Analysis

Data were analyzed using conventional qualitative content analysis [15]. An inductive, data-driven approach was selected due to the lack of existing knowledge and continually developing nature of the pandemic. Participants’ responses often contained more than one idea, so more than one code could be applied to each response. First, two researchers reviewed all survey responses independently, drafted potential code lists, and discussed until a combined coding scheme was developed. Next, two researchers used the coding scheme to code all participant responses. The coders met frequently to discuss, resolve coding differences, and revise the coding scheme. Next, all researchers reviewed the assigned codes and looked for higher-order patterns and groupings, which were designated as themes. The final coding scheme contained 20 codes and 3 themes. A total of 4707 codes were applied. Reflecting the heterogeneity of experiences during the early pandemic, themes represent dimensions which capture a range of positive to negative experiences within them.

## Results

The theme *shock to the (health) system* described sudden changes in healthcare access and larger systems (i.e. healthcare, public health, work places, etc.) that impacted and interacted with RD care and symptoms. The *coping with uncertainty* theme reflected how participants managed to carry on in the face of psychological challenges. *The value of social support while isolated* theme described access to support. 45% codes from responses fell into the shock to the (health) system theme, 21% of codes fell into the coping with uncertainty theme, and 22% codes make up the social support during isolation theme.

### Theme 1: a shock to the (health) system

This theme encompasses codes that relate to social and structural institutions, practices, and attitudes impacting participants’ health and their effect on RD care and symptoms.

#### Inadequate access to quality healthcare

This was the most prevalent code, being present in 645 responses, and it characterized a negative change or expected change in healthcare access or quality as a result of the pandemic. Participants often reported inadequate access to healthcare and mental healthcare, including canceled appointments and/or treatments, changes to insurance coverage, telehealth, and medication shortages.

One participant reported having “less access to ‘non-essential’ care like pain management,” but being “scared about ending up in the hospital from COVID-19, because hospitals are actually kind of a dangerous place for me to be with my RD, like having staff enter my hospital room wearing fragrance (which causes anaphylaxis).” This response demonstrated the rippling effect lacking healthcare access can have on the lives of adults with RD. Not only did this participant lack the access they require for their RD, but it caused distress, another code discussed later on.

Not only were many participants unable to access medical providers, but many were also unable to access their medications and treatments. “It is almost impossible to see my Dr. in person. We have lost supplemental health benefits, so I am not filling all prescriptions.” This participant was forced to forgo their prescriptions because of insurance, and others couldn’t access necessary medicine because of medication shortages, supply chain issues, and pandemic restrictions.

#### Hoping for change in social values

This code included hoping for societal changes, including societal attitudes, change in the government, or change in environmental practices. Participants wished for greater acceptance and/or understanding of individuals with RDs across domains, including society as a whole, on an individual level, or within government institutions. These participants also desired greater acceptance of all people and hoped that this pandemic would highlight the need and benefits of protecting the environment.

Some participants focused on their own RD or symptoms when hoping for a change, such as one participant who said that they hope the pandemic “makes people more caring about immunocompromised people”, but many focused on the needs of the RD community as a whole, such as a participant who said “I think the specific needs of those with RD will be highlighted and better understood.” Responses such as these demonstrate the fierce desire many adults with RD have for others to understand them and their unique struggles, and the hope they have placed on the pandemic to spark such a change.

Others focused on values as a whole, unrelated to RD. These responses largely focused on appreciating others, appreciating simplicity, or appreciating the environment. One participant desired “a change in values: more appreciation of friends and family and creativity and simplicity; less attention on material success.”

#### Appreciation for public health efforts

This code consisted of responses that discuss public health, either current measures, predictions, or desires for future measures. This included following or improving current public health measures, improving public sanitation, planning or preparing for future pandemics, virus research, using masks in non-pandemic times, and better personal hygiene. Participants often desired these changes in public health to have an immediate positive impact on the RD community, such as increased sanitation making it safer for immunocompromised people in public. Others also hoped for better future public health measures because they felt the ones for this pandemic did not adequately protect them or their community.

One of the most common public health responses was a desire for the acceptance of mask wearing to extend beyond the pandemic. For many, this made them feel more accepted and safer, especially if they were immunocompromised. One participant said that they “would like to see people keep distancing and wearing masks during flu season.” For others, masks hid stigmatizing visible differences. “I do think a lot about the irony of having to wear a mask. I often wish I could put a bag over my face so people would not see my [facial] paralysis, and now, I can use a mask without people finding it strange I do.”

Many participants were dismayed when people did not follow current public health measures. One participant said that they have seen “a renewed sense of selfishness. I am disheartened and disgusted at the number of people who refuse to wear a mask.” The people who didn’t follow public health measures added more distress for people with RD. A participant said that they “worry for my family and others who do not practice safe distancing or wear a mask to protect others or their families.”

#### Changes to livelihood

Responses in this code include a change in the participants’ work or responsibilities, accessibility, unemployment, engaging in work as coping, or change in income, savings, expenses, etc. The responses gathered during the pandemic demonstrate how much a RD can impact work and how work, in turn, can affect their RD.

Some participants benefited from changes in work during the pandemic, such as work from home policies and better accommodations. Many found working or attending school from home allowed them to better address their needs and symptoms. One participant, whose RD is a form of sleep disorder, said, “I find myself happier and more productive. My school moving courses completely online allowed me to take a course that I otherwise wouldn’t have been able to take because of my sleep disorder making it difficult to drive and requiring naps.”

Others suffered, either from losing employment/income, or symptoms/stress interfering with their job. As one participant said, “Working from home has been exhausting and hard to set boundaries between work/personal time. This has increased my stress level tremendously.” Another states that they “just returned from Italy where I had medical treatment. I spent all of my savings on my treatment and went into debt. I came home to zero income [because of the pandemic]. I cannot travel to my doctors and therapists. The pandemic has taught me to live one day at a time and to ask for help.”

#### Opportunities to improve healthcare access

This code is defined as a positive change or expected change in healthcare as a result of the pandemic. This includes institutional practices, insurance, pharmaceuticals, mental health, etc. Most of the responses in this code hoped for better healthcare options or practices in the future. Other responses in this code result from better accessibility, usually through telehealth, and accessing healthcare to help with coping.

One of our most common responses for this group is a desire for universal healthcare. As one participant sums up, “health care is a human right and should be available to everyone.” Many participants hope the pandemic “will shine a light on the inequality in healthcare in this country and will bring about universal healthcare.”

While telehealth was very detrimental for some people, as seen in the reduced healthcare access code, for others telehealth was largely beneficial. One participant explains that they “think the positive that came out of this is the easier access to doctors that are far away. I usually have to drive 360 miles for most of my healthcare, but those doctors have finally started doing telemedicine which has helped me greatly.” Another participant hoped it would be “possible (to use telehealth) in the future if too sick to attend in person.”

#### Pandemic conditions exacerbating RD symptoms

Systems-level factors had a downstream effect on participants’ RD symptoms*.* Responses in this code include mention of changes or potential changes in symptoms of the RD due to COVID-19 risk or restrictions. In most cases, the uncertainty, stress, and impacts on healthcare access, caused by COVID affected RD symptoms more than COVID-19 infection itself, perhaps because participants were surveyed quite early in the pandemic.

Limited access to healthcare in particular was one of the largest causes for increased symptoms in our study. One participant had to “take a break from the medication I take daily which I need that to stay awake throughout the day” which in turn “meant I have had to give into my illness for this period and my quality of life is not as good.” Additionally, the new challenges of the pandemic also caused an increase of symptoms in our participants. As one participant stated, “It is harder for me having my kids not able to go to school. Having them home all day and having to do homeschooling has caused my pain level to increase without a break. That has been my biggest challenge.” Another said that they “can’t go to regular physical therapy for pain control, so I’m in more pain.”

#### Disrupted supplies

The least prevalent code in this theme is described as a change in accessing or ordering supplies, including food, housing, water, etc. This code also includes hopes for change in supply chains in the future. Some participants were able to benefit from pick-up or delivery systems, but many struggled to get the supplies they needed. One of the participants mentioned that one of their biggest challenges was that “medications/medical supplies are harder to find or much more expensive.” Another participant said that “my usual use of grocery delivery and curbside pickup went haywire, and was unreliable until recently. Shopping for groceries became extremely difficult due to the number of people suddenly accessing it.” One participant who struggled to fill their prescription said “I take hydroxychloroquine and had a hard time finding a pharmacy that had my medication when I needed a refill.”

### Theme 2: coping with uncertainty

This theme includes codes that address participants’ ability to cope with the pandemic and its impacts. Codes such as distress and resilience address the participants’ coping directly, while other codes addressed facilitators or barriers to coping such as physical activity and nature, and keeping occupied.

#### Stress is not good for my RD

The most common types of distress were stress, worry, frustration, and anxiety. The lack of reliable information, especially about their RD, caused many participants distress. One participant reported a “fear for my well-being given the lack of consistent information, or information on how COVID may affect [RD] patients.” Another said, “it’s caused a great deal of anxiety over possible infection and death.” This distress had many negative effects for our participants, especially for their health. One said that “the stress from this caused multiple ulcer flares.” Another reported the effects of the pandemic “caused greater stress which causes trickle down physical issues and sleep disorders.” Another one states that “the worst thing is the COVID-19 pandemic has caused stress…. and stress is NOT good for my RD… causing falls, and injury.”

#### Resilience learned from being rare

Participants discussed their ability to adapt to challenges, including using gratitude, acceptance, meaning making, focusing on the positive, tenacity, self-advocacy, meditation, determination, etc. Focusing on positive distractions or information was a common way people stayed resilient. One participant represents this method with their focus on “finding positive news and useful information, thinking through processes and battling negative thoughts.”

Another frequent response in our data was for participants to acknowledge the bad, but not let it hold them down. One participant said that they “keep on moving forward. It is what it is. No need to dwell on it” Another focuses on “being the type of person that won’t give up easily. My life has never been an easy one, so I guess I am used to challenges.”

#### Coping through nature and movement

This code was used when a participant described a change in their physical activity, or access to nature, as a result of the pandemic. This included more negative responses like canceled physical therapy appointments, not feeling safe going outside, and gyms being closed. However, this code also included positive responses about using nature and/or physical activity as a way to cope.

The participants who lacked access to physical activity or nature often suffered as a result. One participant “did not have [the] opportunity to regularly walk which has impacted my mobility.” Another had their sport practices canceled so “self care and coping [through] sport was not available, leading to anxiety and upset.”

Those who were able to utilize physical activity and nature often were able to cope better. One participant was able to spend more time outside as a result of the pandemic and said “my husband and I built a hydroponic greenhouse from scrap materials and started a garden. It has been good for physical activity, and it’s relaxing.”

#### Coping by keeping occupied

Some participants kept themselves busy during the pandemic with entertainment and/or hobbies. This form of coping was usually based on the idea of distracting oneself from the pandemic with things they enjoy. “Keeping as busy as I can, doing online courses, doing crafts, too much online shopping (so I have something to look forward to).” Another liked to “watch a lot of mindless TV, preferably comedies, read silly books, and sleep a lot.” Another distracted herself by helping others; “keeping busy sewing and making quilts for others.”

#### Gaining control by keeping a routine

Some participants found it helpful to set a schedule or structured goals. This was usually the result of trying to stay productive while at home, trying to pick up new habits, or trying to utilize a schedule to help manage the symptoms of their condition.

Working from home allowed many of our participants more control over their schedules and could accommodate the needs of their condition more effectively. One participant said that they were “able to work from home some days allowing me to sleep in and nap during lunch or after the workday was over, which ended earlier.”

Others used a routine to help them manage life during a pandemic. “Starting and trying to stick to new habits like taking vitamins with my daily medication, using the Nintendo Ring-fit game [a computer game that uses physical activity as an in-game mechanic] to prevent myself from being too sedentary.”

#### Embracing a slower pace of life

This code involved having or desiring a slower or more relaxing pace to life for themselves or others. Individuals who mentioned a slower pace in their responses either liked that COVID got everyone to slow down, or hoped COVID would encourage people to slow down with their lives. One participant predicted that “people will appreciate simple life and nature and true relationship connections more I think.” Another noticed that “the slowing down and staying home has re-engaged families in spending time together, appreciating what we have and maybe even stopped careless spending” and hoped this would continue post pandemic.

### Theme 3: the value of social support while isolated

This theme included codes addressing sources of support or areas where necessary support was lacking.

#### Family support and strain

A participant’s response was coded as family when they experienced a change in support, distraction, stress, or time spent with family. This was the second most prevalent code, which demonstrates the large impact of family during the pandemic.

One participant stated that “having a solid marriage and a husband who is my rock has been a steadying force. We have ridden out other crises together and have only come out stronger.” Another participant details how their family has been supporting them, “friends and family have been essential to coping with COVID, for six weeks I moved in with a family member that was also isolating and had no visitors and no trips outside the home. Family members brought our groceries and other items.”

As helpful as family members could be, they could also cause harm. One participant, who was immunocompromised, thought they had COVID and their “family members made fun of me for taking it seriously.” They also had trouble getting their family to “understand my reasons for self-isolating. “However, those without family support often fared worse. One participant said “I have no family, no support system. I am in constant pain, struggle to pay for daycare and general bills on my income alone.”

#### The value of companionship support

This code reflected a change in a sense of belonging and shared enjoyment with others. This includes changing relationships with colleagues, friends, boyfriend/girlfriends, healthcare practitioners, others with RD, neighbors, church communities, and pets. One participant noted that their “friends have also really been there for me when I need them.” Another “videoconferenced some friends and played games which [have] been fun. Also, I am a part of some support groups on Facebook. They have held some ‘fellowship’ Zoom calls, which has been refreshing.”

However, without this support, many individuals began to feel isolated and lonely. Another felt “loneliness due to how people have responded to being required to wear a mask in my state. It feels like no one cares if me or people like me are alive.”

#### Craving informational support

This code represents a change in availability or reliability of information. This also includes participants changing the amount of news and/or information they are exposed to, or wanting greater importance placed on information related to science, research, or healthcare. Adopting a “news diet”—limiting the amount of news consumed—was a common practice. Many hoped that the pandemic would be used to further research on RD. One participant said they “hope we will learn how to keep those of us with RDs healthy through things like this.”

Many felt let down by a lack of reliable information about the intersection of COVID-19 and RDs. One participant was especially anxious because of the “lack of solid information about my true level of risk.” Another participant stated that for their coping they “unfortunately use a lot of avoidance techniques. I stay off of social media and rarely visit my support group websites. I go to health authority pages for updates and avoid looking at news articles or watching the news.”

#### Opportunity for experiential empathy

A response was classified within this code if the participant experienced a change in emotional support, or believed that others may be able to understand the experience of having a RD better as a result of the pandemic (also known as experiential empathy). One participant said that it “feels like the rest of the world experienced what it’s like to be chronically ill, and having that understanding from others has been nice. Seeing people recognize that what we go through every day is trauma has been validating and makes me feel less like screaming than I normally do.” Another said that “hopefully, folks will have a better understanding of what it feels like to be isolated and unable to access our world; a reflection of what many of us experience already.” Another said “I think people, in general, are gaining a better understanding through the pandemic of what people with underlying illnesses face on a daily basis. Before this, so many people didn’t understand why I’m late frequently, or why I can’t work, or why I don’t ‘just do [certain things] with my children’, as others may do much more easily. I think the COVID phenomenon builds understanding for populations that previously didn’t understand how much people like us struggle on so many levels, and therefore may build more empathy and compassion moving forward.”

#### Disruptions to and opportunities for instrumental support

This code was used when a participant experienced a change in availability of tangible support for activities of daily living, including housekeeping, caregiving, home maintenance, governmental support, etc. For the majority of our responses in this code, it was a loss of support. However, a few participants did have better support during the pandemic. One participant was “quite happy that many stores have started offering curbside pickup. I have a great deal of trouble shopping at garden centers because they are so warm, so having a curbside pickup has actually encouraged me to shop more at the garden center. I also have started using the curbside at the grocery store and will probably continue to do this once the pandemic is over.”

#### Helping others cope

The smallest code included responses where the participant described providing support to others. Participants helped others by volunteering for a RD support group or organization. For example, one participant “continued significant volunteer responsibilities, including leading state-wide Zoom meetings, trainings, political advocacy, media interviews.” Others aimed to fill a need they saw around them, like one participant who spent their time “making activated charcoal filtered masks for my family and friends, and sharing of scientific information about the spreading and how to avoid it.”

## Discussion

This study is unique in that it captured the psychosocial experiences of adults with RDs in the early stages of the COVID-19 pandemic. During the first wave of the pandemic, the major experiences of people with RDs included a shock to the (health) system, coping with uncertainty, and the value of social support while isolated. Narratives from these participants provide evidence and context to support previous research about the unique challenges the RD community faces [6], as well as the new challenges this pandemic caused [13]. While the pandemic caused obstacles for nearly everyone, our study supports that adults with RDs faced additional challenges, particularly with healthcare access, stress, and a lack of social support. This study offers valuable insights on making improvements for the RD community, both in and outside of a pandemic.

The largest concern from participants was inadequate access to quality healthcare. This took many forms from canceled appointments and treatments, to challenges getting prescriptions and medical supplies. These results align with the healthcare access challenges found in the few previous studies of people with RDs during the pandemic [8, 9, 11–13]. This disruption caused waves of impact throughout their lives, affecting symptoms, mental health, work, activities of daily living, and social connection. However, participants noted silver linings in healthcare, some of which were noted in previous work [12, 13]. In particular, expanded telehealth enhanced their ability to access medical and mental health providers and RD specialists (which previously required significant travel) [7, 12]. A unique finding was that participants hoped for future advances like the application of COVID-19 science to develop new RD treatments.

Importantly, our study makes a unique contribution to the RD COVID-19 literature by examining psychosocial factors. During the pandemic, most participants derived their support from family and friends while other support methods that participants had previously relied on were disrupted. Those with sufficient support often did not experience the same stress, uncertainty, feelings of isolation, and inability to complete daily tasks as those who lacked support. These findings are supported by previous research demonstrating better outcomes in the RD community as a result of support, such as a study finding that emotional and companionship support are associated with satisfaction with life amongst individuals with RDs [16].

While the majority of our participants’ responses concerned negative experiences, there were some positive psychosocial experiences and expectations that arose as well. Work from home policies especially seemed to benefit many participants, who reported being able to manage their condition more effectively. Participants also hoped that the current pandemic would improve public health measures, both in and out of pandemics. There was also a great desire for societal changes. They hoped the pandemic would generate experiential empathy among people without RDs or disabilities so that they would better understand what it is like to live with a poorly understood illness. Participants wanted society to be more accepting and accommodating of the unique needs of RDs, and they hoped for better policies to address the unique challenges this community faces. Participants hoped that COVID-19 would highlight current health inequities experienced by people with RDs and other minorities and inspire the changes they so deeply desire.

Like many, participants experienced significant distress due to the pandemic. However, some of this was amplified due to having a RD, including a lack of reliable information about how COVID-19 might interact with their RD. Yet, participants displayed resilience and a variety of coping strategies, including focusing on the positive and meaning-making. Many participants said they were used to challenges due to their RD, and that had given them the strength to persevere. Several kept busy by providing support to others. Many people with RDs appreciated that COVID-19 slowed the pace of life, offering opportunities to rest, appreciate what they have, and re-engage true relationship connections.

The aspects of our study concerning coping, support, and silver linings are largely missing in the current RD literature. As such, our findings shed light on the opportunities revealed by the pandemic. Our findings foreshadow systemic improvements that could be made to reduce social and health disparities for people with RDs, including more robust public health policy, mask wearing, and sanitation, government policies and/or benefits ensuring flexible work options and access to supplies, and acceleration of healthcare funding, research and treatment.

### Strengths and limitations

As is (regrettably) common in RD research [12, 13], our sample overrepresented people who identify as white, female, who are middle aged, and middle class. People with these demographics likely had more resources compared to the true population of the RD community, which means the experiences represented in our study may be more positive than the reality for many.

Our data was collected during the first spring and summer of the pandemic, representing a unique moment early in the pandemic when some of the greatest uncertainty occurred. The effect of this uncertainty is seen throughout our data and offers a snapshot of arguably the most difficult time from the pandemic. As several researchers on our team have RDs, our insider experiences in the community allowed us to formulate questions and understand themes that researchers without lived experience may not have generated.

## Conclusion

As noted in previous literature, those with RDs often face unique challenges, and this proved to be especially true during COVID. The shock to the healthcare system imposed by the pandemic exacerbated the challenges of having a RD. Participants struggled to cope with uncertainty, but some felt their experience with RD prepared them to be resilient. Findings also demonstrated the importance of having support through such unprecedented times and the consequences that result from not having it. Many participants were hopeful that COVID-19 could inspire the changes they so desperately wanted. They hoped that the social and health inequities highlighted by the pandemic, especially those affecting individuals with RDs, will finally be addressed and greater respect and understanding will be had for everyone. They also hope that emphasizing the flaws in public health and healthcare systems will prompt the changes needed to improve these systems.

### Supplementary Information

**Additional file 1.** Interview questions.

## Data Availability

The data used for the current study are not publicly available in order to protect participant confidentiality. The open-ended responses analyzed for this study may contain identifiable information. The data are available from the corresponding author on reasonable request. To protect participant confidentiality, Institutional Review Board and/or Ethics board approval is required.
