# Supplementary material for: A shock to the (health) system: experiences of adults with rare disorders during the first COVID-19 wave
Source: Orphanet J Rare Dis. 2024 Jan 30;19:33. doi: 10.1186/s13023-024-03033-z (PMC10829377; doi:10.1186/s13023-024-03033-z)
Supplement: Supplementary file 1 — Additional file 1. Interview questions. [file 13023_2024_3033_MOESM1_ESM.docx]

**Open-ended questions**

- How has the COVID-19 pandemic impacted your life with a rare disorder?
- What are difficulties you're experiencing during COVID-19?
- What has helped you cope with and manage challenges that have come up related to COVID-19?
- Has the pandemic affected your ability to receive healthcare for your rare disorder?
- Has there been any change in your access to healthcare services during this period and if so, how has this changed?
- What barriers are you experiencing to managing your health and well-being during this period?
- What is helping you manage your health and well-being effectively during this period?
- Do you think anything positive will come out of the COVID-19 pandemic for you or others with rare disorders?
- Are there any cultural or societal changes that you would like to come out of the COVID-19 pandemic?
